# The Inherent Coupling of Intrinsically Disordered Regions in the Multidomain Receptor Tyrosine Kinase KIT

**DOI:** 10.3390/ijms23031589

**Published:** 2022-01-29

**Authors:** Julie Ledoux, Alain Trouvé, Luba Tchertanov

**Affiliations:** Centre Borelli, ENS Paris-Saclay, CNRS, Université Paris-Saclay, 4 Avenue des Sciences, F-91190 Gif-sur-Yvette, France; julie.ledoux@ens-paris-saclay.fr (J.L.); alain.trouve@ens-paris-saclay.fr (A.T.)

**Keywords:** receptor tyrosine kinase (RTK), full-length KIT cytoplasmic region, intrinsically disordered regions, phosphotyrosine, modelling, molecular dynamics, conformational transition, allosteric regulation, free energy landscape

## Abstract

RTK KIT regulates a variety of crucial cellular processes via its cytoplasmic domain (CD), which is composed of the tyrosine kinase domain, crowned by the highly flexible domains—the juxtamembrane region, kinase insertion domain, and C-tail, which are key recruitment regions for downstream signalling proteins. To prepare a structural basis for the characterization of the interactions of KIT with its signalling proteins (KIT INTERACTOME), we generated the 3D model of the full-length CD attached to the transmembrane helix. This generic model of KIT in inactive state was studied by molecular dynamics simulation under conditions mimicking the natural environment of KIT. With the accurate atomistic description of the multidomain KIT dynamics, we explained its intrinsic (intra-domain) and extrinsic (inter-domain) disorder and represented the conformational assemble of KIT through free energy landscapes. Strongly coupled movements within each domain and between distant domains of KIT prove the functional interdependence of these regions, described as allosteric regulation, a phenomenon widely observed in many proteins. We suggested that KIT, in its inactive state, encodes all properties of the active protein and its post-transduction events.

## 1. Introduction

Receptor tyrosine kinases (RTKs) are cell surface receptors with a highly selective affinity to numerous ligands—growth factors, cytokines, and hormones. Each RTK acts as a sensor for its specific extracellular ligand, whose binding triggers dimerization of the receptor, activation of its kinase function, and auto-phosphorylation of particular tyrosine residues in the cytoplasmic domain [1]. This mechanism leads to the recruitment and activation of multiple downstream signalling proteins, which carry the signal to the nucleus, thus altering the patterns of the gene transcriptions governing various aspects of the cell physiology. Initiation of this cascade-like process involves different regions of multidomain RTKs, each of them acting in a finely concerted behaviour, mediated by a tightly regulated allosteric mechanism controlling all their physiological processes [2,3]. The explicit elucidation of the signalling cascades represents a critical and unsolved problem in cell biology.

**Figure 1:**
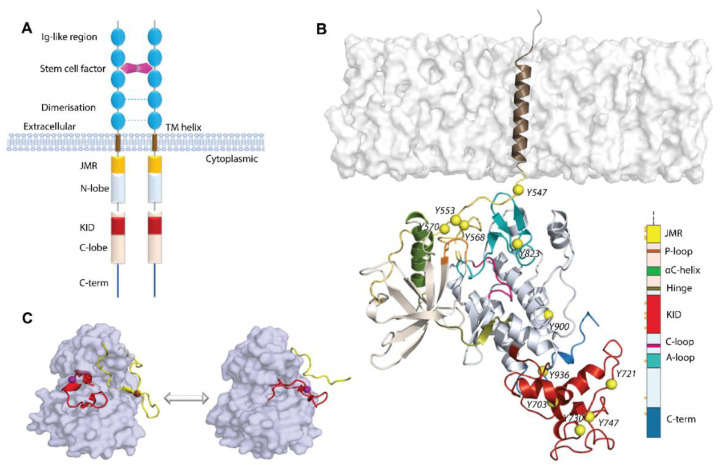
Structure of RTKs, illustrated on KIT, a member of the RTK family III. (**A**) Structural composition of KIT: an extracellular domain (ED) with five Ig-like regions, a transmembrane domain (TM helix), and a cytoplasmic domain (CD), composed of the juxtamembrane region (JMR), N- and C-lobe, spliced by a kinase insert domain (KID), and C-tail domain. The stem cell factor (SCF) extracellular binding induces dimerization and activation of KIT. (**B**) Structural model of the KIT containing CD and TM helix. The protein is shown as cartoon, membrane as grey surface, the phosphotyrosine residues (Y) as yellow balls. The KIT regions are coloured as shown on the scheme. (**C**) The inactive-to-active state transition of KIT is shown using the crystallographic structures of KIT CD in the inactive (PDB: 1T45) and active (PDB: 1PKG) states. In the inactive state (left), JMR (in yellow) is in the auto-inhibited conformation, stabilized through contacts with A-loop (in red), αC-helix, and C-loop. A-loop is packed to the TK domain. Both regions, JMR and A-loop, protect the catalytic site from ATP binding. In the active state (right), JMR and A-loop move from the TK domain to a solvent exposed position and deploy out of the active site, allowing ATP to access its binding site. The protein is shown as the solvent accessible surface, with JMR and A-loop as cartoon.

Each RTK, depending on the location of its different domains, is composed of an extracellular domain (ED) and cytoplasmic domain (CD), linked by a single transmembrane helix (TM) (Figure 1A). In turn, each domain of RTKs has a modular architecture consisting of several structural blocks, interconnected by coiled linkers providing high conformational plasticity. The ED, highly variable in RTKs, is formed by diversified units (Ig-like, cysteine-rich, and cadherin fragments [1]) containing the highly selective ligand-binding site, while the CD architecture is similar and usually composed of the juxtamembrane region (JMR), bi-lobe tyrosine kinase (TK) domain with an ATP-binding region (N-lobe), and phosphotransferase domain (C-lobe), linked by a loop (hinge). In some RTK families, the canonical TK domain is interrupted by a kinase insert domain (KID) [4].

The ligand-induced stimulation of RTKs promotes conformational changes of the ED, governing its dimerization (except IsulinR) and the signal transmission from the extracellular environment to the intracellular area. The conceptually straightforward mechanisms for the ligand-induced dimerization of RTKs are surprisingly different and encoded primarily by the sequence and structure of the extracellular domain [1,5]. As the structures of the intracellular domain of RTKs in the inactive state are distinct, the activation mechanism is specific for each receptor. As for the structures of the active state, they are rather similar, in which key regulatory elements, including the ‘activation loop’ (A-loop) and αC-helix in the kinase N-lobe, adopt a particular configuration in all activated TK domains that is necessary for catalysis and phosphotransfer reaction [6,7] (Figure 1C).

The numerous studies focused on the regions containing tyrosine residues—JMR, A-loop, KID, and C-terminal—put in evidence their roles in different steps of RTK activation. For instance, in several RTKs, FLT3 [8], KIT [9], and the EPH family [10], the inactive state of the TK domain is maintained by the auto-inhibited configuration of JMR, which is stabilised by extensive contacts with residues of the TK domain, including A-loop. The role of A-loop in kinase activation is not conserved in RTKs, e.g., the A-loop tyrosine is not necessary for EGF receptor activation [11], whereas its phosphorylation is essential for the activation of the insulin receptor [12,13].

KIT, one of 58 human RTKs, is activated by the binding of a growth factor, the stem cell factor (SCF), and regulates a variety of critical cellular processes, such as proliferation and differentiation, cell survival and metabolism, cell migration, and cell cycle control [14,15]. The aberrant activation of KIT inducing deregulation of signalling networks is associated with the progression of many cancer types, including human acute myeloid leukaemia, aggressive systemic mastocytosis, melanoma, gastrointestinal stromal tumour, and stomach cancers [16,17,18]. Disclosure of the KIT-activated pathways in carcinogenesis will be a crucial step towards the development of KIT-targeted therapies [19].

The human KIT is composed of 976 amino acids, in which residues L525–Y545, K546–K581, W582–S931, and T932-R946 represent the TMD, JMR, TK domain, and C-tail, respectively. In response to SCF binding, KIT activates several signalling pathways, using its rich set of phosphorylation sites localised on different fragments, i.e., JMR, A-loop, KID, C-lobe, and C-tail, which are the docking sites (scaffolds) of numerous proteins. In KIT, eight tyrosine phosphorylation sites have been identified in vivo (Y568 and Y570 in JMR; Y703, Y721, and Y730 in KID; Y823 in A-loop; Y900 in the C-lobe; and Y936 in C-tail) [20], as well as two additional sites detected in vitro in the activated kinase domain (Y547 and Y553 in JMR) [21].

As expected from the critical role of JMR in the regulation of KIT kinase activity, two tyrosine residues, Y568 and Y570, are involved in the maintenance of the auto-inhibited conformation that blocks the regulatory αC-helix and ATP-binding P-loop, thereby suppressing kinase activity [9,22]. Upon SCF-stimulated activation, JMR adopts the solvent-accessible position, and these two tyrosine residues, Y568 and Y570, are the first to be phosphorylated and involved in downstream signalling [23]. Both phosphorylated tyrosine residues identified in vivo act as docking sites for signalling molecules with Src 2 homology domains (SH2), which, in turn, transmit the signal further through the cell [24].

The A-loop Y823 is likely to play a role of a pseudo-substrate, interacting with D792 in C-loop and, thus, maintaining the inactive conformation of KIT. It was reported that phosphorylation of Y823 is not required for KIT activation [21]; however, this residue is crucial for cell survival and proliferation [25]. The function of Y823 in post-transduction processes is still not clear, and it may interact with signalling proteins, but such interaction partners have yet to be identified [26]. The functions of JMR and A-loop appear to be strongly coordinated, not only in the inactive state of the KIT (auto-inhibition function), but also during KIT activation, when their concerted departure from the auto-inhibited positions in the inactive state contributes to other conformational changes in the protein, e.g., the removal of αC-helix and P-loop from their positions in the inactive state.

It is widely accepted that KID does not influence the kinase activity of KIT [27], and its functional role is to provide alternative binding sites for adaptors, signalling, and scaffolding proteins in the cytoplasm, through five functional phosphorylation sites, three tyrosine (Y703, Y721, and Y730), and two serine (S741 and S746) [28]. The C-terminal tail, containing phosphotyrosine residue Y936, also contributes directly to intracellular signalling [29]. Phosphorylation of KIT at Y703 and Y936 activates the mitogen-activated protein kinase (MAPK) pathway [30]. CrkII was identified to specifically bind Y900 in a phosphorylation-dependent manner, possibly via the p85 subunit of PI3-kinase [31].

Consequently, the different KIT regions regulate the catalytic process and events that activate and control the signalling cascade. As such, KIT functions are dependent on more than one region, and these regions should be directly or collaterally coupled. The study of interconnections between different, distant, or adjacent KIT regions, at the structural and dynamical levels, may shed light on their cooperativity required for different KIT functions.

Such study is pertinent because the 3D model of the nearly complete cytoplasmic domain of KIT, in which the empirically (X-ray crystallography) determined kinase domain, completed by the de novo KID and C-tail, was reported [32]. To study the interconnections between the functional region, i.e., JMR, TK domain, KID, and C-tail, the full-length cytoplasmic domain of KIT was investigated in its native environment, with a fully reconstructed JMR, attached to the transmembrane (TM) helix and inserted into the membrane (Figure 1B). The study was performed by conventional molecular dynamics (cMD) simulation, which generates atomic-level data of intrinsically high resolution.

We suggested that such a level of description of KIT, with the fully reconstructed JMR, KID, and C-tail, will elucidate the structural and dynamical properties of its different functional regions that contain (or not) the phosphotyrosine residues. Such characterisation can more explicitly explain the role of each region in maintaining KIT inactive state and establish the relationships between the regions showing either their cooperation or autonomy.

## 2. Results

### 2.1. Data Generation and Proceeding

The 3D model of the KIT CD possessing KID and C-tail [32] was completed by the JMR segment T544-W557 and transmembrane helix attached to JMR. The multidomain construct of 431 amino acids (I516-R946) was studied by conventional MD simulations (cMD and all-atom, with explicit water and membrane) as a membrane protein. The extended MD simulation was repeated three times (replicas 1–3, each of 2 µs, started with different randomised initial atomic velocities and performed upon strictly identical conditions) to extend conformational sampling and examine the consistency and completeness of the produced KIT conformations. Each simulation started with an equilibrated conformation, obtained after minimising the neutralised solvated model. The generated data sets were analysed for a full-length construct and per domain/region. To avoid the motion of the protein as a rigid body, all data were normalised by least-square fitting of MD conformations to the initial conformation (t = 0 μs) as a reference.

### 2.2. General Characterisation of MD Conformations

The root-mean-square deviations (RMSDs), computed for each conformation, respective to the same initial conformation, display comparable profiles in all cMD trajectories, demonstrating the good reproducibility of the generated data (Figure S1). As shown by the per-domain analysis, the large variability of the RMSDs, calculated for all Cα-atoms of KIT, is mainly impacted by JMR, KID, and C-terminal, while the RMSDs of the TK N- and C-lobes show high stability. Similarly, the profile of the root-mean-square fluctuations (RMSFs) curves is comparable in the three MD trajectories, with differences only in the amplitude of the RMS fluctuations of the N- and C-terminals and KID. It is also interesting to note the increased RMSD and RMSF values for A-loop in trajectory 2.

### 2.3. 2D Folding and 3D Structure of KIT in Inactive State

**Figure 2:**
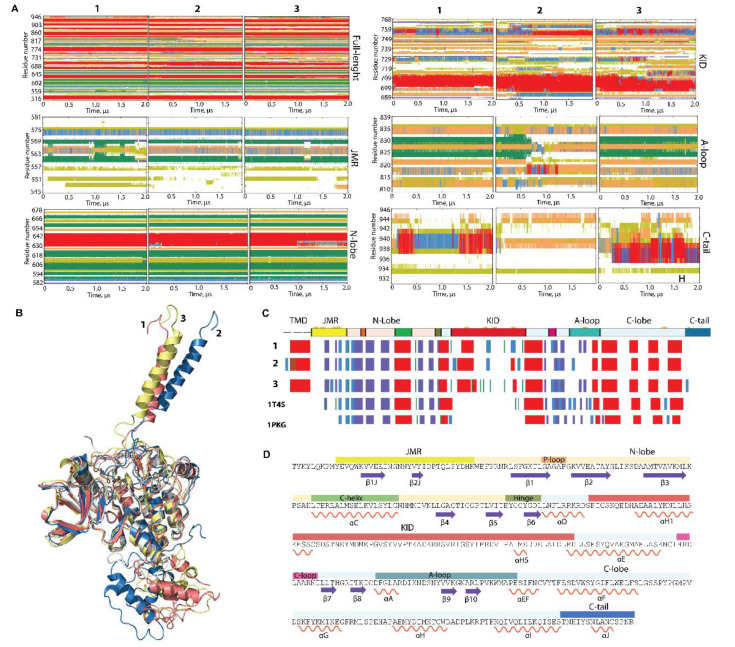
Folding of RTK KIT. (**A**) The time-related evolution of the secondary structures of the entire, full-length KIT and per domain/region, as assigned by the define secondary structure of proteins (DSSP) method [34]: α-helices in red, 3_10_-helices in blue, parallel β strands in green, antiparallel β strands in dark blue, turns in orange, and bends in dark yellow. The three cMD replicas (1–3) were analysed individuially. (**B**) The 3D structure of KIT is shown by superimposition of the final conformation of the TK domain (t = 2 µs) of each trajectory. (**C**) The secondary structures—αH- (red), 3_10_-helices (light blue), and β-strands (dark blue)—assigned for a mean conformation of every MD trajectory (1–3) and the crystallographic structures 1T45 and 1PKG. (**D**) The secondary structures—αH- (red) and β-strands (dark blue)—assigned on the mean conformation of the concateneted trajectory are labelled as in [35].

The secondary structure interpretation of KIT conformations indicates that the folding is generally well-conserved in the TK domain and corresponds perfectly to those in the crystallographic structures 1T45 (inactive state) and 1PKG (active state), while JMR, KID, and C-tail changed their folding during and between each trajectory (Figure 2). The intrinsic disorder of KID was previously characterised [32,33], and we suggest that JMR and C-tail are also intrinsically disordered regions (IDRs).

Indeed, the repeated conversion of two 3_10_-helices (I563-N566 and D572-Q575) into turn or bend, as well as the partial instability of two β-strands, are good arguments to classify JMR as an IDR. Similarly, the alteration of α-, 3_10_-helices, bend, turn, and coil in C-tail proves its disordered nature. A-loop also displays a noticeable transformation of its fold, evidenced as a reversible transition of a turn to a 3_10_-helix in three distinct A-loop segments, and a full unfolding of the antiparallel sheet, as observed in trajectory 2 (Figure 2A). Surprisingly, the secondary structures of αC-helix, considered early as a canonical structure that only changes its position upon the activation [9,36], was slightly perturbed in some conformations by decrease of its size.

According to these observations, the multidomain KIT comprises at least four ID regions—JMR, KID, A-loop, and C-tail—centred around the structurally stable core, the TK domain. The TK domain, together with its secondary structure stability, exhibits weak or very weak inherent dynamics, as viewed by the small values of RMSD, RMSF for the residues from this domain (Figure S1), and limited angular variations (≤10°) of αC- and αE-helix, the representative fragments from the N- and C-lobe, respectively (Figure S2).

**Figure 3:**
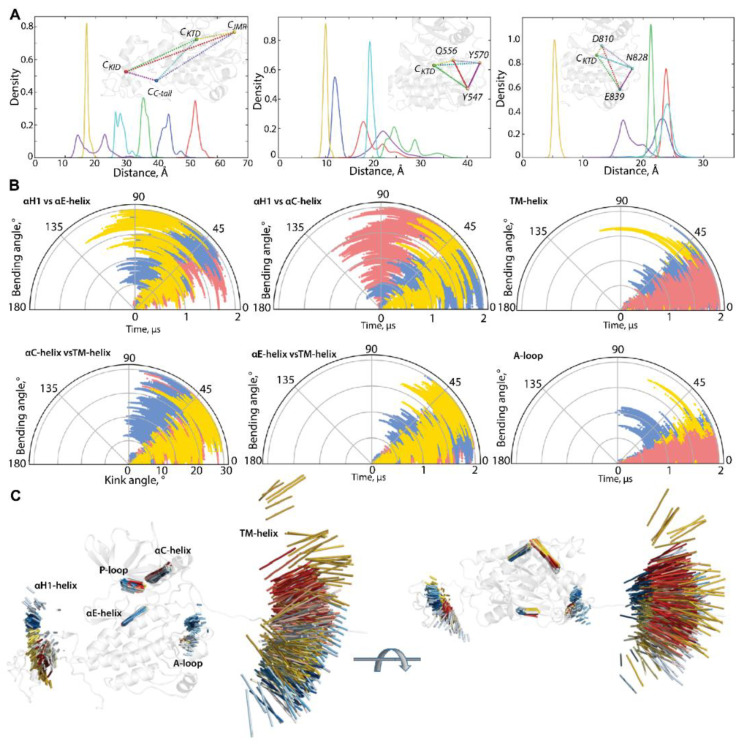
Geometry of KIT conformations from the cMD trajectory. (**A**) Geometry of the tetrahedrons with nodes designed on centroids (**C**) of the KIT domains or the C-atoms of residues, as shown on inserts. (**B**) Curvature between the KIT helices, TM helix, and curvature of the β-hinge of A-loop. Calculations are performed after least-square fitting of the data on the kinase domain. Conformations from different trajectories are distinguished by colour: red (1), blue (2), and yellow (3). (**C**) Positions of hints—TM-helix, αC-helix, αE-helix, αH1-helix, P-loop, and β-hinge of A-loop—each taken 10 ns, are superimposed on the mean conformation of KIT, calculated on the concatenated data. The protein is shown as cartoon, each hint presented by an axis of helix or vector collinear with a β-strand. Two orthogonal projections are shown. All calculations are performed on cMD conformations, each taken 10 ps from the trajectories distinguished by colour—red (1), blue (2), and yellow (3) in (**B**,**C**). The colour gradient shows the evolution of a trajectory, from light (t = 0 µs) to dark (t = 2 µs).

The great variability of the RMSD and RMSF values of KIT apparently derived from the two distinct factors, i.e., (i) an unsteady position of the flexible KID, JMR, and C-tail, with respect to the relatively stable TK domain, (ii) the intrinsic properties KID, JMR, and C-tail, connected to their unstable (metastable and transient) folding and/or conformational changes. Each KIT region can be regarded, in a first approximation, as a pseudo-rigid body with its local centre of gravity (centroid, C). Centroids, determined on JMR (C_JMR_), KID (C_KID_), C-tail (C_C-tail_), and the TK domain (C_TKD_), are the nodes of a dynamical tetrahedron that reflects the displacement of each region, respective to one another (Figure 3A).

The particularly large range of C_KID_∙∙∙C_C-tail_ distances with two well-resolved and considerably distant apexes at 12 and 23 Å and reflects the distinct position of the C-tail, with respect to KID, effect that was observed in [32]. Using the data generated during three 2-µs cMD simulations, we found that C-tail is positioned in proximity to KID and C-lobe, at approximately equal frequency—in 40 and 46% of conformations, respectively. The other conformations (14%) are positioned between these two border locations.

A low variance of distances between the centroids of KID and the TK domain (34–38 Å) suggests that the different positions of KID, derived mainly from its turn round (twist), relative to the TK domain and not from a linear displacement. As the αH1-helix is the most structurally stable element of the disordered KID [33], it can be assigned as a hint (reference) for the characterisation of the KID position in the KIT. The KID displacement relative to the TK domain was measured with the distance between the Cα-atoms of Y703 (αH1-helix) and Y774 (αE-helix), as well as the bending angle defined by the vectors coinciding with the αH1- and αE-helix axes. These metrics describe, in a first approximation, two main types of motion—linear (translation) and angular (rotation).

During the first 0.5 µs of simulations, the distance between the tyrosine residues of αH1- and αE-helix varies slightly around their mean values (mv of 25–27 Å), while the bending angle between the αH1- and αE-helix shows quite different angular positions, from parallel to orthogonal (Figure S2 and Figure 3). Obviously, the main movement of KID, relative to the TK domain, is rotation, while its linear displacement is rather limited. The measured metrics, distance, and bending angle do not correlate with each other.

The distance between the centroids of TKD and JMR (C_TKD_∙∙∙C_JMR_) is a very approximate metric to estimate the position of the long and flexible JMR, composed of multiple segments—the JM-Proximal (JM-P), JM-Buried (JM-B), JM-Switch (JM-S), and JM-Zipper (JM-Z)—functionally specific and, in the 3D structure of KIT, are largely distant [15]. To improve the accuracy of the characterization of the JMR positions, we calculated the distance between the TK domain (C_TKD_) to the selected residues of each JMR segment—Y547 (JM-P), Q556 (JM-B), and Y570 (JM-S)—except for JM-Z, which is adjacent to the TK domain N-lobe, while maintaining its position (Figure 3). The distances between JM-P and C_TKD_, and JM-P and the other JMR fragments, show the most variations which suggest more than one JM-P positions, a typical manifestation of structural disorder. Likewise, the geometry of the tetrahedron, determined on C_TKD_ and the residues of the extended A-loop, shows a large displacement of the β-hairpin (represented by N828), while the two ends of A-loop (calculated on D810 and E839) changed slightly. Displacement of the β-hairpin, with alternating secondary structures and a wide range of A-loop curvature, reveals heterogeneous conformations of A-loop, indicating its partial disorder.

The transmembrane (TM) helix conserves its perfect α-helical structure but displays a change in its orientation in space. To find out if pivot spaces were preferred, the distribution of the bending and kink angles [37] were obtained (Figure 3). We found that the TM helix pivots within a space defined by a cone with an apex angle of 45°. Such movement of the TM helices is frequently observed for membrane proteins in a crowded environment [38,39].

The geometry of the multidomain cytoplasmic region of KIT can be described as relative to the structurally conserved TK domain, through the bending angles between the representative fragments, taken as hints. The bending angles show the abundant diversity of the TMD orientation, in respect to the TK domain, which is richer for the N-lobe (TM-helix versus αC-helix) than the C-lobe (TM-helix versus αE-helix) (Figure 3B). As was expected, most variations of the bending angle are observed for KID.

Finally, the positions of the selected hints (TM-helix, αC-helix, αE-helix, αH1-helix, P-loop, and β-hinge of A-loop), superimposed on the mean conformation of KIT, show their relative dynamical positions during the cMD simulations, reflecting the generic geometry of KIT (Figure 3C and Figure S3).

### 2.4. Inter-Domain, Non-Covalent Interactions of Kit in Inactive State

We have suggested that the positions of JMR, KID, and C-tail, observed in KIT conformations, are controlled by non-covalent interactions, primary by H-bonds. This hypothesis is based on the analysis of non-covalent contacts stabilising the crystallographic structure of the kinase domain of the inactive KIT [9], which evidenced that the abundance of H-bonds is the most important factor providing the directional interactions that underpin the protein structure (Figure S4). Intra-domain interactions mainly contribute to the stabilisation of the β-sheet in N-lobe (H-bonds) and coiled-coil structure in the C-lobe (principally, hydrophobic forces).

**Figure 4:**
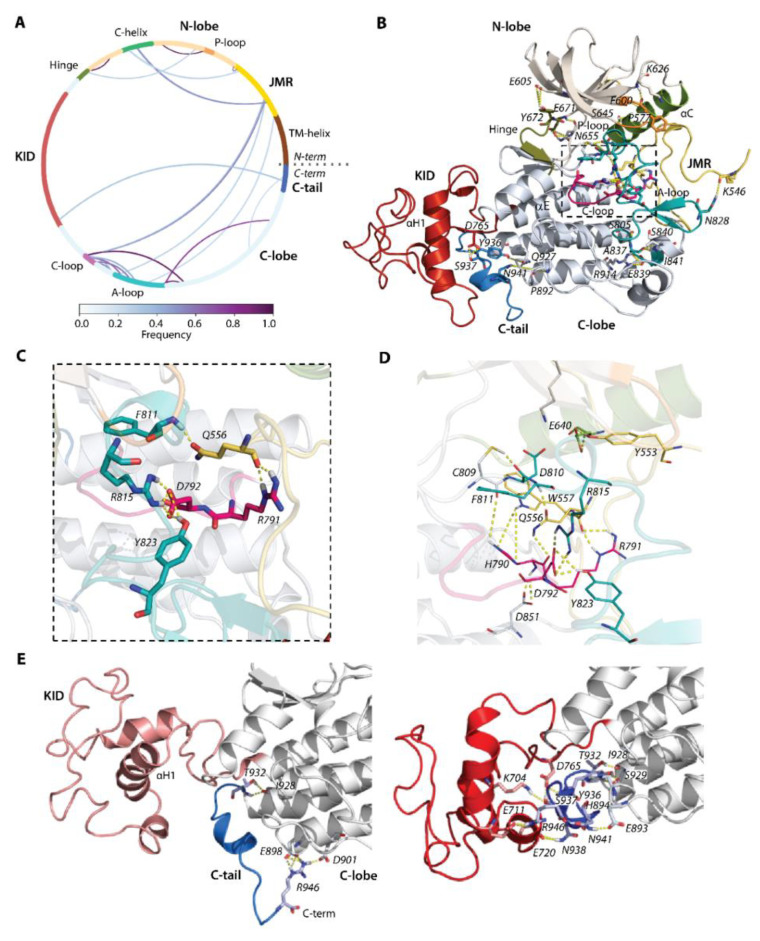
Hydrogen bonds stabilizing the inactive state of RTK KIT. (**A**) The cords diagram compiles the H-bonds of multidomain KIT, shown as curves, coloured according to the occurrences, from 0 (white) to 100% (violet). The H-bonds (yellow dashed lines), shown on 3D structure of KIT (**B**), are zoomed on the active site (**C**) and active site with neighbour residues (**D**). (**E**) H-bonds stabilizing C-tail at the TK domain (left) and at KID (right). (**A**–**E**) The protein is shown as cartoon, in which the domains and functionally related fragments are distinguished by colour and labelled in bold and regular font, respectively. Residues contributing to H-bonds are shown as sticks and labelled in italic; the H-bonds are shown as yellow dashed lines. Calculations are performed on the concatenated trajectories 1–3.

For our analysis, in each MD conformation of KIT, the H-bonds involved in the formation of regular structures (helix or sheet) and those contributing to the intra-domain framework (e.g., the β-sheet and helix motifs) were excluded. The remaining H-bond contacts represent an H-bonding pattern of multidomain KIT that displays the interaction between distinct domains (Figure 4). In terms of protein topology, the H-bond pattern represents interactions between residues that are either close-positioned in the sequence (e.g., A- and C-loop) or distant (e.g., JMR and A-loop) but are neighbours in three-dimensional structure. In terms of strength, these interactions are strong and long-lived (regular) or correspond to weak and unsteady contacts. Some contacts, both regular and rare, are multiple and frequently represent bifurcated H-bonds or involve pairs of adjacent residues forming side (parallel) interactions.

By focusing on the functionally significant regions contributing to the activation/deactivation mechanism of KIT, we found that the extended JMR forms multiple and regular H-bonds, involving its different segments interacting with distinct fragments of kinase domain—the JM-Proximal segment (JM-P, T544–D552) with the antiparallel sheet of A-loop (H-bond K546∙∙∙N828), JM-Buried segment (JM-B, Y553−V559) with αC-helix (H-bond Y553∙∙∙E640), JM-Zipper (JM-Z, residues D572−K581) with the αC-helix (H-bond P577∙∙∙S645), and JM-Switch (JM-S, V560−I571) with C- (H-bond W557∙∙∙H790 and bifurcate H-bond H790∙∙∙Q556∙∙∙R791) and A-loop (H-bond Q556∙∙∙F811). Similarly, the catalytic (C-) and activation (A-) loops are tightly coupled by the salt bridge (D792∙∙∙R815) and H-bonds D792∙∙∙Y823. The position of the P-loop in the N-lobe of the kinase domain is maintained by the H-bond P600∙∙∙K626.

This extensive H-bond pattern of KIT performs crucial functions—(i) maintaining the JMR in its auto-inhibited position [9], (ii) retaining F811 in the active site (the DFG motif, D810-F811-G812) [36], and (iii) coupling of D792 with two residues of A-loop R815 and Y823 that ensures the allosteric communication between A-loop and JMR [40]. Further stabilisation of A-loop in the packed position is provided by the negatively-charged flanking residues from its ends, forming the regular H-bonds with the residues of the C-lobe—the catalytic aspartate of the DFG motif forms H-bond D810∙∙∙C809, and glutamate constitutes a salt bridge with R914 (E839∙∙∙R914). The hinge between N- and C-lobe is involved in multiple H-bonds with each lobe. From one side, it interacts with a twisted β-sheet of five antiparallel strands of the N-lobe (Figure 4B), as well as from the other side its β-strand (β7) is linked with β-strand (β8) of C-lobe, forming the stable antiparallel inter-lobe β-sheet (not shown). Likewise, the inter-domain H-bonds contribute to C-tail stabilisation. As we observed, the cMD conformations are grouped into two large clusters, with respect to the relative positions of C-tail, KID, and C-lobe (Figure 3A). Except the H-bond, I928∙∙∙T932 observed in conformations of both clusters, the conformations of these clusters display two binding modes that hold C-tail, either at KID or adjacent to C-lobe (Figure 4D).

In particular, the C-tail localised at proximity to KID interacts simultaneously with charged residues of KID and C-lobe, serving as a bridge between these two KIT domains (Figure 4E). For the interaction with KID and C-lobe, C-tail uses alternative sets of residues. C-tail at C-lobe is maintained by the multiple H-bonds of its R946, with the charged residues E898 and D901 in C-lobe. These interactions are probably false, due to the N-terminal status of R946. Phosphotyrosine Y936 does not appear to be involved in H-bonds. Similarly, the primary phosphorylation sites Y568 and Y570 (identified in vivo) from JMR apparently do not contribute to the KIT interaction network and are largely exposed with their OH moieties to the solvent. One of the two additional phosphorylation sites found in vitro (Y553) is involved in the interaction with αC-helix through its sidechain (Figure 4B,D). Likewise, Y823 of the A-loop interacts with the sidechain carboxyl of D792, stabilising the A-loop in close proximity to the C-loop and making D792 unavailable for any catalytic process.

**Figure 5:**
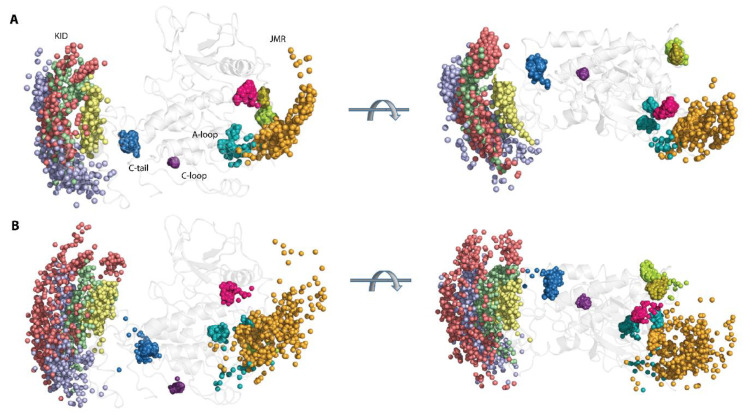
Geometry of the tyrosine residues in KIT. The spatial distribution of the Cα-atoms from the tyrosine residues (**A**) and their hydroxyl groups (OH), presented by the oxygen (O) atoms (**B**), is shown in two orthogonal projections with the coloured Cα- and O-atoms: Y547 in orange, Y553 in magenta, Y568 in smith green, Y570 in lime, Y703 in yellow, Y721 in lilac, Y730 in red, Y747 in green, Y823 in teal, Y900 in violet, and Y936 in blue. The Cα- and O-atoms positions were extracted from the MD conformations, taken each 10 ns, fitted on the TK domain of the initial structure (t = 0 ns), and superimposed on this structure (countered cartoon in grey).

The position of the phosphorylation sites in the KIT structure strongly depends on their guest fragments from their conformational features and relative position in KIT. Thus, the large displacement (mainly rotational) of KID from the TK domain is reflected in the expanded distributions of the Cα-atoms position and hydroxyl groups of Y721, Y747, and Y730, located on the highly flexible fragments of the disordered KID, while the OH groups of Y703 in the stable αH1-helix form the narrower cluster (Figure 5).

Likewise, the compact distribution of the Y553, Y568, and Y570 locations, viewed by the Cα-atoms and the OH groups, reflect the stable position of JMR-B, JM-Z, and JMR-S, while the wide-ranging distribution of the Y547 location corresponds either to the multiple inherent JMR conformations or ample displacement of JM-P, with respect to the TK domain. The three clusters of the unique phosphotyrosine of A-loop, Y823, apparently reflect three different conformations of A-loop.

### 2.5. KIT Structure Per Domain, Internal Motion, and Intra-Domain Interactions

To characterize the inherent structural and conformational properties of the variable KIT regions, i.e., JMR, KID, A-loop, and C-tail, and estimate the contribution of these properties to the structural and dynamical relationships of KIT, each of these fragments was analysed individually. First, the conformations of each fragment were grouped by ensemble-based clustering [41] using different RMSD cut-off values, varying from 2.0 to 5.0 Å, with a step of 0.5 Å. Using a cut-off value of less than 4 Å results in many poor-populated clusters, while a cut-off value of 4 Å was sufficient to regroup the conformations of all fragments into clusters that give the best cumulative population (>95%; Figure S5).

**Figure 6:**
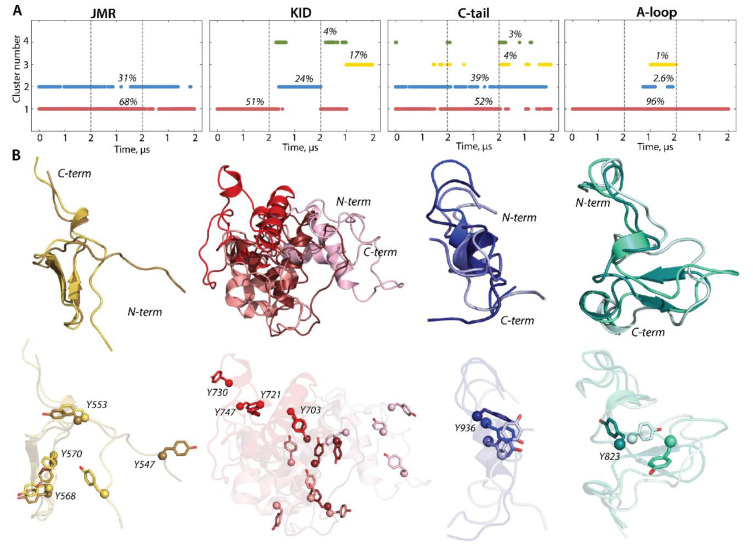
Structure and conformation of the disordered fragments of KIT—JMR, KID, A-loop, and C-tail. (**A**) The clusters of conformations, obtained by ensemble-based clustering (cut-off 4 Å) and their population. (**B**) Superimposed representative conformations from the clusters. The protein is shown as cartoon, with the tyrosine residues as sticks. The colour gradient shows the population of clusters, from dark (most populated) to light (less populated). Calculations were carried out on cMD conformations, taken every 100 ps from the concatenated trajectories after the fitting on the initial conformation of the TK domain (at t = 0 µs).

The majority of JMR conformations forms the two most populated clusters, C1 (68%) and C2 (31%), composed of the conformations observed in each cMD trajectory (Figure 6). All JMR conformations show similar secondary structures, a short β-sheet in JM-S segment, and a transient 3_10_-helix in JM-Z, regularly undergoing reversible folding–unfolding events. The representative conformations of the most populated clusters, 1 and 2, differ only in the position of the JM-P segment containing Y547, which was identified in vitro as a phosphotyrosine [21]. Such alternative position of JM-P leads to a large area of the Y547 location, while the other tyrosines are almost superimposed (Figure 5 and Figure 6).

The conformations of the intrinsically disordered KID, previously characterised in [33], were grouped into four clusters. Two clusters, C2 (24%) and C3 (17%), are composed of conformations generated over the alone trajectory, 2 and 3, respectively, while the most populated cluster C1 (51%) and small cluster C4 (4%) are composed of conformations from at least two independent trajectories. The representative conformations from the distinct clusters differ at the folding level (2D) and in 3D structure organization, reflecting a high level of intrinsic disorder in KID. The ample rotation of KID, with respect to the TK domain and its large conformational diversity, leads to dispersed location of the tyrosine residues.

The conformations of C-tail are grouped in the two most populated clusters, C1 (52%) and C2 (39%), and two lowly populated clusters, C3 (4%) and C4 (3%). The representative conformations of C1, C2, and C3 show similar secondary structures, described as an extended random coil with a small transient helix in the middle (α-helix↔3_10_-helix), and differ mainly by the orientation of the C-terminal residues. All clusters regroup conformations generated over the three independent trajectories, and, apparently, C-tail secondary structures do not influence a cluster separation. Indeed, the small transient helix is only observed in trajectories 1 and 3, while the conformations from trajectory 2 are folded as a random coil (Figure 2 and Figure 5). The tyrosine residue Y936 shows a close position and orientation of its OH group in most conformations (1–3 clusters) and is different in the only rare intermediate conformations.

Focusing on A-loop, we noted that the most populated cluster (C1, 90%) is composed of conformations containing a transient β-hairpin (β-hairpin↔turn/coil). The lowly populated cluster (C2, 2.6%) is formed by the coiled A-loop, with a small transient helix (α-helix↔3_10_-helix↔turn) and tinny cluster C3 (1%) groups conformations with a random coil structure. The alternative position of Y823, found in different conformations of A-loop analysed individually (Figure 6), is represented by three distinct clusters, which show the Y813 position in KIT (by location of its Cα atom and OH group) (Figure 5).

### 2.6. Intrinsic Motion in KIT and Its Interdependence

The intrinsic dynamics of the multidomain KIT was first analysed with the cross-correlation matrix, computed for the Cα-atom pairs of the full-length protein. As the matrices calculated for the three MD trajectories are very similar (Figure S6); therefore, we will discuss only one, randomly chosen, matrix map.

**Figure 7:**
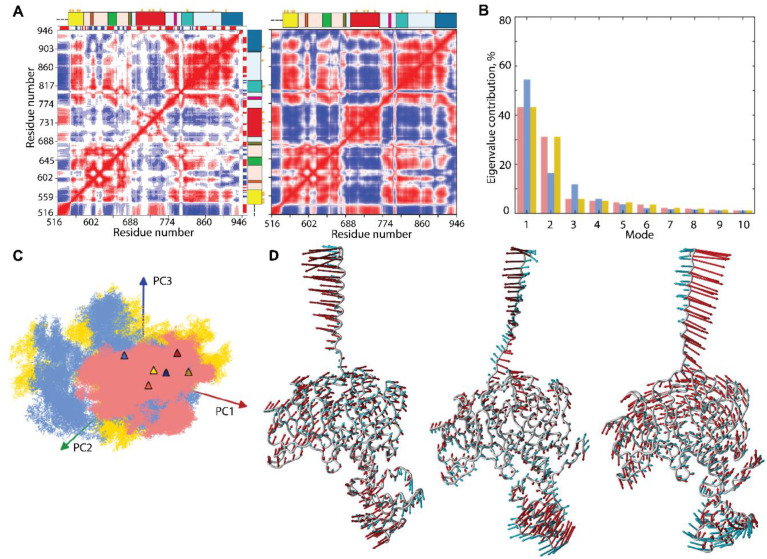
Intrinsic motion in KIT and its interdependence. (**A**) Dynamical inter-residue, cross-correlation map, computed for the Cα-atom pairs of MD conformations (left) and resulting from NMA (right) of KIT. The displayed results represent trajectory 1. Correlated (positive) and anti-correlated (negative) motions between Cα-atom pairs are shown as a red-blue gradient. (**B**) PCA modes calculated for KIT after least-square fitting of the MD conformations to the mean conformation. The bar plot gives the eigenvalue spectra, in descending order, for the first 10 modes calculated on cMD trajectories 1–3 (left). (**C**) Projection of the KIT cMD conformations onto the first two modes, calculated with principal component analysis (PCA) (right). MD trajectories 1, 2, and 3 denoted in red, blue, and yellow, respectively. Light and dark symbols display the first and the last conformations for each trajectory. (**D**) Atomic components in PCA modes 1–2 are drawn as red (1st mode) and cyan (2nd mode) arrows, projected on the cartoon of KIT. A cut-off distance of 4 Å was used.

The Cα–Cα pairwise, cross-correlation map demonstrates highly coupled motions within each KIT domain and between the structural domains, even largely distant (Figure 7A).

The patchwork pattern in N-lobe reflects the positively correlated motion of the seven stands in the crossed β-pleated sheet and their coupling with αC-helix. Similarly, the helices of C-lobe are mutually correlated (positively), forming a map of well-defined blocks, distorted by A-loop. Both TK lobes correlate negatively with the KID and TM helix and positively with JMR. C-tail correlates positively with C-lobe and negatively with N-lobe. The inter-lobe correlations are not homogeneous, and, apparently, P-loop and αC-helix of N-lobe play a specific role in the motion correlations. The motions of TM helix and JMR are contrariwise.

Such correlation patterns can be partially explained by the overall architectural features of the studied KIT, which has a strongly extended shape. Movements of one end (TM helix) are counterbalanced by movements of the opposite end (KID) to provide a stable balance of KIT around its centre of gravity. On the other hand, the highly coupled motion in KIT may reflect the allosteric effect(s) and has the functionally related content—regulation function. In both cases, the cross-correlation pattern indicates the strongly coupled movements of the largely distant domains/regions and reflects the block-segmented movement of the multidomain KIT. The similar pattern, even more pronounced and contrasted, was observed on the cross-correlation matrix (Figure 7), calculated using the normal mode analysis (NMA), calculated on the mean KIT conformation of each trajectory with force field for Cα-atoms, developed by [42]. Moreover, the NMA cross-correlation maps are very comparable for the three mean conformations and calculated using different force fields [43,44], showing a great reproducibility of the results (Figure S7).

The collective motions of KIT, characterised by PCA, showed that two ten modes describe ~70–80% of the total fluctuations of the multidomain KIT (Figure 7B). The two first modes clearly reflect a highly coupled motions of the multidomain KIT. The great mobility of the TM helix is gradually increased from its *C*- to N-ends. The amplitude and direction of motion of the TM helix and KID differ in the three trajectories (Figure 3 and Figure 7D), suggesting a larger conformational space for the KIT than was observed in each trajectory, probably larger than the total space of all trajectories. The TK domain global motions in KIT demonstrate a lower amplitude, with respect to that of TM helix and KID, as that the motions of all TK residues are collective and may be described as a circular pendulum-like movement along a common virtual rotational axis. Interestingly, the virtual axis is coincident (centred) with the active site of KIT.

The projection of the cMD conformations on the first two PCA modes revealed that (i) the conformational density is extended in **1** and **2** trajectories, as well as a more compact in replicate **3**; and (ii) the superimposed KIT conformations of the three cMD simulations provide a wide coverage (overlap) of PC subspaces (Figure 7C).

### 2.7. Conformational Space of KIT and Its Representation as Landscape

Since the RTK KIT contains at least four disordered fragments, showing reversible protein folding–unfolding events and a large conformational diversity, its conformational space can be represented more explicitly as a ‘free energy landscape’ model. Such interpretation of the intrinsically disordered multidomain protein leads to quantitatively significant results, allowing the comparison between different states. The relative Gibbs free energy, ΔG, defined on chosen coordinates called ‘reaction coordinates’ or ‘collective variables’, describes the conformations of a protein between two or more states, measured as the probability of finding the system in those states. Such representations of protein sampling, with the use of reaction coordinates, can be the quintessential model system for barrier crossing events in proteins [45].

**Figure 8:**
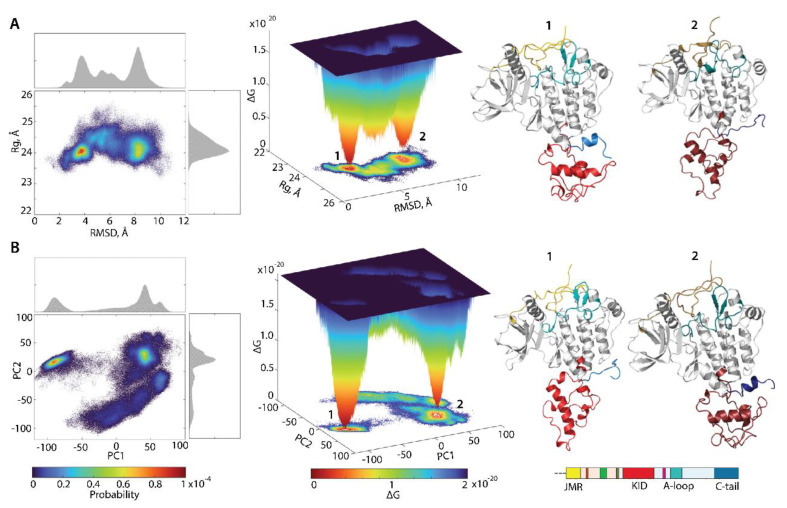
Free energy landscape (FEL) of KIT as a function of the reaction coordinates, (**A**) Rg  (in Å) versus RMSD (in Å) and (**B**) two PCA components (PC1 versus PC2), was generated on the MD conformations of KIT for the conformational ensemble, sampled on the merged replicas and fitted on the initial conformation taken at t = 0 µs. (Left column) The two-dimensional representation of FEL of the KIT conformational ensembles. Density distribution of each reaction coordinate is shown at the top and right, respectively. (Middle column) The three-dimensional representation of the relative Gibbs free energy. (Left and middle column) The red colour represents high occurrence, yellow and green represent low, and blue represents lowest occurrence. The free energy surface was plotted using Matlab. (Right column) KIT conformations from wells 1 and 2.

For the evaluation of the relative free energy (ΔG) and reconstruction of its landscape, the distant measures—radius of gyration (Rg), distance (RMSD), and the PCA components (PC1 and PC2)—were used as reaction coordinates for the description of the ΔG landscape of KIT. The free energy landscape (FEL), as a function of RMSD and radius of gyration Rg (${\mathrm{FEL}}_{\mathrm{RMSD}}^{R_{g}})$, as well as the PCA components (${\mathrm{FEL}}_{\mathrm{PC}1}^{\mathrm{PC}2})$, calculated for the concatenated replicas of KIT, is shown in Figure 8.

Each FEL shows a rugged landscape, reflecting a high conformational heterogeneity of the multidomain KIT. This heterogeneity adds complexity to the interpretation of the free energy map and limits the detection of spontaneous state-to-state transition, when using the conventional sampling method (cMD). Nevertheless, both FEL of KIT show well-defined areas of minimum energy indicated in red, which represent more stable conformations (the thermodynamically more favourable state), while the reddened areas indicate conformational transitions of the protein.

The unimodal Gaussian distribution of $R_{g}$ does not separate the KIT conformations on isolated clusters but delimits the most populated region; the ΔG minima, showed by lower energy values, are rather defined by the multimodal distribution of RMSDs. The ${\mathrm{FEL}}_{\mathrm{RMSD}}^{R_{g}}$ of KIT has two depth minima, separated from each other by energy barriers of various heights, shown as an additional smooth local minimum. Regarding the ${\mathrm{FEL}}_{\mathrm{PC}1}^{\mathrm{PC}2}$, this local smooth minimum is absent because the free energy landscape, reconstructed on the principal components, only represents the conformations reflecting the ‘essential’ dynamics of protein, which constitutes two almost iso-energetic minima. As the first two PCs only represent movements on a larger spatial scale (larger spatial scale motions), so that the energy landscape, defined on these reaction coordinates, ${\mathrm{FEL}}_{\mathrm{PC}1}^{\mathrm{PC}2}$, loses smaller spatial scale motions (e.g., intermediate conformations).

Nevertheless, these FELs, reconstituted on two different sets of reaction coordinates, showed that the ensemble of KIT conformations can recover the two-state picture characterized by two global minima (wells); each of them is composed of similar conformations, which are very distinct between the two minima (Figure 8). The composition of these wells is permuted, due to the different reaction coordinates which lead to the permuted population of two minima: the conformations of the first and second minima on ${\mathrm{FEL}}_{\mathrm{RMSD}}^{R_{g}}$ corresponds to the content of the second and first minima on the ${\mathrm{FEL}}_{\mathrm{PC}1}^{\mathrm{PC}2}$. The KIT conformations from the two global minima in both landscapes display large conformational alteration in KID and C-tail, which appears to be the main factor in such a two-state landscape.

The low approximate population of conformations in the two deepest wells on the free energy landscape of KIT (16 and 7% on the ${\mathrm{FEL}}_{\mathrm{RMSD}}^{R_{g}}$ and 12 and 11% on the ${\mathrm{FEL}}_{\mathrm{PC}1}^{\mathrm{PC}2}$) indicated that the great number of KIT conformations are the intermediate between these states. We suggested that the estimation of the contribution of each domain to the total energy landscape of the multidomain KIT (the per-domain relative free energy ΔG) will complete the reconstructed landscape of the protein. Each domain was considered individually, but as a dependent subdomain of the entire KIT; therefore, the corresponding data were fitted on the initial conformation (t = 0 µs) of the full-length of the cytoplasmic region of KIT.

**Figure 9:**
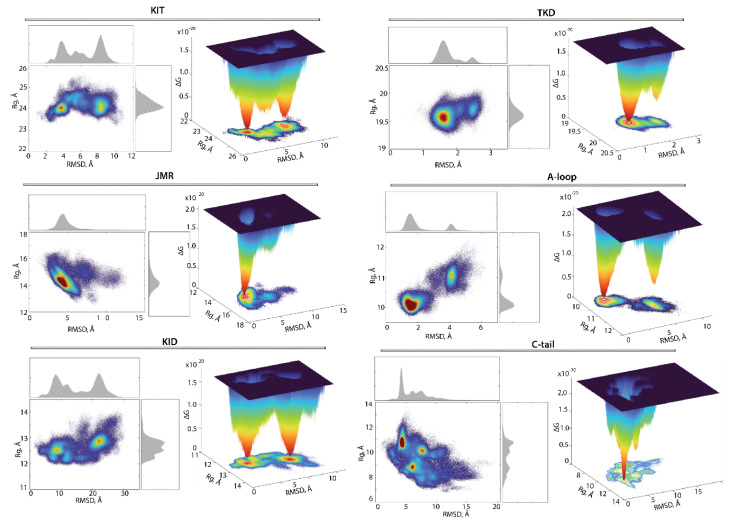
Free energy landscape (FEL) of KIT and its subdomains, as a function of the reaction coordinates, Rg (in Å) versus RMSD (in Å). The FELs were generated for each entity using the ensemble of MD conformations, sampled on the merged replicas and fitted on TK domain. (**Left**) The two-dimensional representation of FEL. Density distribution of each reaction coordinate is shown at the top and right, respectively. (**Right**) The three-dimensional representation of the relative Gibbs free energy. The red colour represents high occurrences, yellow and green represent low occurrences, and blue represents the lowest occurrences. The free energy surface was plotted using Matlab.

As was expected, the tyrosine kinase domain showed only one highly populated (54%) and deep well completed by similar conformations, while the second well, if it still exists, is considerably reduced and contains 4% conformations (Figure 9). The second well may be composed of the KIT showing alternative conformations of its structural fragment, e.g., A-loop. Indeed, the two-state profile of A-loop, with unevenly filled contents of the wells unequally (40 and 4%), is in good agreement with the profile of the TK domain, which confirms our hypothesis.

Surprisingly, the FEL determined on the JMR showed only one highly populated deep well (47%). By comparing this observation with the ensemble-based clustering of JMR (Figure 7), its impact on the total free energy landscape can rather be attributed to its alternate position, relative to the tyrosine kinase domain, than as an effect of its internal conformational features.

The free energy landscape of KID differs from that reported in [33] and was apparently biased by the false movement of JMR. Indeed, in KIT with the highly flexible JMR with the N-terminal status, the unique global energy minimum of the KID, accompanied by the two local minima showing higher energy values, was observed [33]. The free-energy landscape of KID from KIT, composed of the cytoplasmic domain linked to TMD (present study), shows two deep wells with an almost equivalent population (15 and 14%), supplemented by a series of satellite wells with the essentially lower population (from 0.5 to 2.5%). Such a two-main state profile of KID is presumably the main factor contributing to the two-state profile of KIT.

The FEL profile of C-tail shows unique low energy deep minimum, which is complemented by two wells with the higher energy values. As C-tail conformations have similar secondary structures and differ mainly in the orientation of the C-terminus. These intrinsic properties do not contribute significantly to the FEL, which is primarily impacted by the alternate location of the C-tail, with respect to KID and/or to TK domain.

## 3. Discussions

Remarkably, the multidomain modular RTK KIT consists of a quasi-stable TK domain, crowned by at least four intrinsically (inherently) disordered (ID) domains/regions—JMR, KID, A-loop, and C-tail. These disordered regions contain functional tyrosine residues and act as a ground platform for the recruitment of signalling proteins. The implication of disordered regions in post-translational modifications is well-known [46,47,48]. Nevertheless, despite their fundamental role in mediating the signalling cascade, the intrinsically disordered domains of KIT (and other RTKs) are critically under-studied, leaving us with a naïve, over-simplistic, and rather schematic view of these domains as structurally impersonal chains of varying lengths, enabling conformational adaptability to ensure constitutive attachment of specific protein partners.

This paper focuses on the functionally significant domains in KIT signalling and their disorder, described at two levels, such as the intra-domain intrinsic disorder (the local disorder) and extrinsic disorder (inter-domain), observed at the KIT level.

Since KIT ID domains have no single, well-defined 3D structure, they cannot adequately be described as simple statistical coiled or helically folded chains equally populating all MD conformations allowed by their backbone torsion angles. Instead, KIT ID domains contain transient, short- and long-length structures, which display various degrees of compaction and elongations. Therefore, each ID domain of KIT is not homogeneous, but represents a very complex mixture of a broad variety of differently folded conformations, ranging from the partially folded to fully unfolded, which, in turn, are foldable. In KIT, these regions are mainly composed of polar and charged residues, while the portion of hydrophobic residues is reduced, presenting an archetypal sequence composition of the intrinsically disordered proteins [49,50].

The ID regions of KIT belongs to two types—the very elongated (extended) and poorly folded regions (JMR, A-loop, and C-tail), as well as the globule-like (collapsed) KID, with a high level of the helical structures.

The high content of unfolded residues (random coil) in JMR and A-loop implies a potentially high degree of disorder in these regions. However, since these regions, in the inactive state of KIT, are attached to the TK domain by numerous H-bonds, which anchor each end of A-loop and bind the JMR along most of its length, their disorder is limited. Only the short segments of these regions, not stabilized by the H-bonds, show the local disorder, which is evidenced by the alternating positions of JM-P on JMR and the unfolding of the β-hinge in A-loop. Since the disordered segment of JMR contains the functional tyrosine Y547, this residue is highly dislocated in 3D space over a wide range and, therefore, is accessible for phosphorylation events, even in inactive KIT or its intermediate state. Y823 in A-loop is also irregularly positioned in space, located in at least in three different positions, but its side chain (hydroxyphenyl) is still oriented towards the active site of KIT.

We suggest that, upon activation of KIT, induced by SCF binding, JMR displaced from its packed auto-inhibited position will achieve the most extended and flexible conformation, so that its level of disorder will increase, and, therefore, the level of its adaptability required for scaffolding (docking sites) and recruitment of different protein binding partners will also increase.

The position of Y936 and orientation of its phosphorylable sidechain is highly conserved in all conformations of the extended C-tail, regardless of the location of its disordered C-end, either near KID or close to the TK domain. It may well be that these results do not fully reflect the role of the C-end; in our model, it was shortened, with respect to the native length.

The higher portion of charged and polar residues in KID, compared to JMR and A-loop, is reflected in the enhanced disorder of KID, which involves almost all residues of the domain, except of a single helix (αH1), which shows stable folding (secondary structures) but varies greatly in its orientation, respective to the TK domain. The structure of the αH1-helix is conserved, not only in the KID fused to the tyrosine kinase domain but also in KID simulated as a cleaved polypeptide [33].

The KID disorder is manifested first as transient folding (secondary structures) and presented as a collection of highly variable helices, permanently converting between α- and 3_10_ helices, as well as between a helix and non-regular structure (turn, bend, and coil), whose length and type change. Second, as KID helices are connected by flexible linkers of varying length, depending on the order of the helical folding, this promotes many relative orientations of the helices, leading to a large set of very heterogeneous conformations. These two factors, transient folding and high conformational flexibility, are the main items characterizing the intrinsic intra-domain (local) disorder of KID. Nevertheless, as we have reported [33], the intrinsically disordered KID acquires a globular shape, stabilized by non-covalent interactions—H-bonds and hydrophobic forces. Hydrophobic contacts are compiled, as a well-organised hydrophobic core maintaining KID compactness.

Moreover, this compact globule-like domain is displaced (linear and angular displacement) as a ‘pseudo-rigid’ body, with respect to the TK domain, such representing the inter-domain extrinsic disorder. This movement is also highly heterogeneous, both in space and time, with a variable contribution of the linear and angular components, manifested in the form of small dislocations and large-scale rearrangements. This all-scale movement can involve distinct segments of KID, again showing the highly anisotropic nature of the intrinsic disordered in this domain.

The two-level disorder (intrinsic and extrinsic) provides the high conformational variability and adaptability of JMR, KID, and C-tail required for the scaffolding (docking sites) and recruitment of different protein partners of KIT and facilitates the regulation of cellular processes. The overall structure of KIT represents a continuous spectrum of conformations, with a different degree and depth of disorder, as was reported for the other functional proteins [49,51], thereby generating a complex protein structural space that defines a structure–disorder continuum, with no clear boundary between ordered and disordered proteins/regions. The disorder of at least four regions of the multidomain KIT is reflected in its free energy landscape, which lacks a unique global deep minimum that can be found in ordered proteins and appears as two local minima joined/separated by a ‘flattened plateau’ containing the intermediate conformations.

Classically, multiple binding events in ID KIT, regulated by phosphorylation, can be characterized as binary on/off switches. However, it has been reported that phosphorylation can generate more complex responses [52], and multi-site phosphorylation can additionally generate sensitive threshold responses, as well as graduated responses. Therefore, successive phosphorylation events can additively modify (increase or decrease) the binding affinity, allowing for graduated responses, or they can modulate the conformational set, with an impact on signalling output.

Such simplified and flattened energy landscape has shown that KIT is extremely sensitive to different environmental changes (e.g., phosphorylation) that can alter its free energy landscape in different ways, e.g., lowering some energy barriers, while raising certain energetic minima. This explains the conformational plasticity of ID regions, allowing them to evolve faster than protein domains that adopt defined stable structures, and its ability to interact with several different partners and, therefore, fold in different ways.

Since ID domains are multiple in RTK KIT, we asked, does the disorder/order of one domain depend on the disorder/order of other remote regions? In other words, we wish to understand whether the folding–unfolding process of the intrinsically disordered domains in inactive KIT is orchestrated or not. Does the allosteric regulation of KIT involve the disordered domains/regions?

Highly coupled motions between distant sites of KIT, as evidenced by the cross-correlation maps, suggests its association with the functional dependence of these regions, which is classified as allosteric regulation, the phenomenon largely observed in many proteins [2,3,53,54]. In particular, the coupling motions in each lobe, N- and C-lobe, of the TK domain and between the lobes reflect the allosteric regulation of the kinase function, which is well-described for different non-receptor and receptor tyrosine kinases [3,55,56]. The coupled motions of two activating regions of KIT, A-loop, and JMR were characterized, in terms of their allosteric communication in the wild-type KIT, which was disrupted in oncogenic mutants [40,57].

The mechanism of regulation of the RTK regions directly involved in cell signalling is still a matter that is little studied, due to the absence of structural data characterizing these regions.

To the best of our knowledge, we have presented, for the first time, a model of a full-length cytoplasmic region of an RTK KIT attached to a transmembrane helix and its molecular dynamics simulations, under conditions that mimic the natural environment of the KIT. We demonstrated the tight coupling between the KIT remote regions populated by phosphotyrosines (JMR, KID, and C-tail), which serves as a scaffold for the recruitment and activation of signalling proteins.

The classical molecular models, the Koshland-Nemethy-Filmer [58] and Monod-Wyman-Changeux [59] paradigms describing the allostery, ‘a second secrete of life’ [60,61], take, as a physical basis, the conformational changes between well-defined structural states of proteins but do not consider other factors, such as conformational dynamics, monomeric-oligomeric states, intrinsic disorders, and negligible conformational changes [62]. Recent empirical observations, demonstrating that allostery, can be facilitated by dynamic and intrinsically disordered proteins [2,63,64,65] and have resulted in a new paradigm to understand allosteric mechanisms, which focuses on the conformational ensemble and statistical nature of the interactions responsible for the transmission of information [64,66].

The manifestation of allostery in intrinsically disordered proteins (IDP) is one of the most sophisticated phenomena observed in the last decade [3,50,51,67,68,69]. The conformational dynamics of folded structures and large-scale disorders are important for allostery [64,70], but the quantitative understanding of this phenomenon remains a great challenge. It is not easy to understand and describe the allosteric phenomenon with a common point of view, encompassing both highly structured and disordered systems. Moreover, it was suggested that the intrinsic disorder of the RTKs renders not only infer flexibility and high accessibility of binding sites, but certain chain dimensions and spatial organizations may influence the organization of the signalling complexes, orchestration of protein interactions and, in the end, signalling outcomes [46]. Following this concept, instead of considering the ID domains of KIT as passive scaffolds for its protein partners, we put forward a more complex view of active orchestration via organizational and operational features left uncovered within their disorder. Moreover, we suggest that all properties of the activated RTK KIT and post-transduction events, initiated by the active KIT, are encoded in its inactive state.

## 4. Methods

### 4.1. Modelling

***The full-length cytoplasmic domain of KIT in the inactive state***. The 3D structure of the inactive RTK KIT (K558-R946) was taken at 2 μs of molecular dynamics (MD) simulation, as reported in [32]. Structure of the missing JMR fragment (T544-W557) was retrieved from the PDB structure 3G0E (resolution: 1.6 Å) [71]. Five thousand (5000) models of the full-length cytoplasmic domain of KIT (T544-R946), in the inactive state, were generated for the human sequence P10721 (https://www.uniprot.org/uniprot/, accessed on 17 June 2021) using Modeller 10.1 [72] and the available structural data. The best model was chosen according to its DOPE score [73] and stereochemical quality (Procheck) [74].

As the generated N-terminal of KIT was not accessible for its linking to the transmembrane domain, the alternative conformations were calculated by the normal mode analysis (NMA) from the R library bio3D [75]. Fifteen (15) modes were calculated at 310 K, with all available force fields. The seventh mode, calculated with the All-atom Elastic Network 2 (aaenm2) force field, showed the greatest fluctuations. As the fragment T544-W557 is rigidified by the strong H-bond T544∙∙∙K547, a short (1 ns) MD simulation (in water solution) of the inactive KIT was performed, upon constrains to remove this H-bond. The model of KIT, with relaxed N-terminal end (KIT^T544-R946^), allowed the latter modelling steps.

***The full-length cytoplasmic domain of KIT in the inactive state linked to the transmembrane helix.*** The 3D model of the transmembrane α-helix (L521-L543), created with the Builder module of PyMOL 1.9 (http://www.pymol.org/pymol, accessed on 17 June 2021), and last conformation of KIT^T544-R946^, from the 1-ns MD simulation, were used as templates for construction of the full-length cytoplasmic domain of KIT, in the inactive state, with its transmembrane helix (KIT^L521-R946^).

Five thousand (5000) models of KIT^L521-R946^ were generated with Modeller 10.1 [72] using the human sequence (P10721). To avoid the sticking of the transmembrane domain to the cytoplasmic domain during the minimisation procedure, refinement was performed only on H517-L521 and M541-Y545. The best model was chosen according to its DOPE score [73] and stereochemical quality, assessed with Procheck [74].

***The full-length cytoplasmic domain of KIT with transmembrane helix inserted into membrane.*** A phosphatidylcholine (POPC) lipid bilayer was generated using Charmm-Gui [76]. As a single transmembrane α-helix was not found in the Orientation of Protein in Membrane (OPM) database [77], the orientation of the double-helix of PDGFR-β, a cousin of KIT, was considered. Suggesting that the single helix in monomer of KIT can have an alternative orientation, KIT^L521-R946^ was oriented manually, so that its transmembrane helix was positioned perpendicularly to the bilayer with polar residues, next to the phospholipids’ polar heads and apolar residues among their tails. Finally, to reduce the number of residues located in the extracellular area, the N-terminal extremity of KIT was reduced to I516-R946 (KIT^I516-R946^).

### 4.2. Molecular Dynamics Simulation

***System set-up*.** Each system, KIT^T544-R946^ and KIT^I516-R946^, wrapped in a 23 Å-width leaflet lipid bilayer of POPC (2), were solvated with TIP3P water model in a rectangular box, with the PACKMOL-Memgen [78] and LEaP modules of AmberTools20 (http://ambermd.org/AmberTools.php; accessed on 17 June 2021), using the ff14SB all-atom force field [79] for protein and Lipid17 for membrane: (i) hydrogen atoms were added; (ii) protonation states of amino-acids at physiological pH were assigned, as well as the histidine residues protonated on their ε-nitrogen atoms; (iii) no counter-ions were added, as the system is already of neutral charge. The systems, KIT^T544-R946^, in the water solution (1), and KIT^I516-R946^, wrapped in a 23 Å-width leaflet lipid bilayer of POPC (2), contained 71,062 atoms in total, with 6415 atoms of protein and 64,647 atoms of water (1), as well as 172,368 atoms in total with 6869 atoms of protein, 42,074 atoms of the lipid membrane, and 123,423 atoms of water (2).

***Minimisation, equilibration and data generation***. The systems were equilibrated using the Sander module of AmberTools20. For system (1), 30,000 minimization steps were performed, i.e., (i) 10,000 on the all-atom fixed protein to relax the water, (ii) 10,000 with fixed Cα atoms to allow the relaxation of sidechains, and (iii) 10,000 without any constraints on the system. For (2), the positional constraints of various forces were applied and subsequently decreased at each minimisation/equilibration step to allow a smooth equilibration. The values were for: all protein atoms—10, 10, 2.5, 1, 0.5, and 0.1; the phosphate atom of POPC—2.5, 2.5, 1, 0.5, and 0.1 kcal/mol; the dihedral angle around the double bond of the oleoyl chain of POPC (restricted to 0°) and dihedral angle formed by the glycerol carbon and the oleoyl ester oxygen atoms of POPC (restricted to 120°)—250, 250, 100, 50, and 25 kcal/mol. The system (2) was minimised during 5000 steps (2500 steps of steepest descent then 2500 steps of conjugated gradient).

For both systems, the following steps were executed. A 100 ps thermalisation step was performed, where the temperature (atoms velocity) was gradually increased from 0 to 310 K using the Berendsen thermostat [80]. Then, a 100 ps equilibration step with a constant volume and 100 ps equilibration step with constant pressure (1 bar) were performed. Periodic boundaries conditions and isotropic position scaling were imposed with the Berendsen barostat [80]. For these two steps, temperature regulation was performed with Langevin dynamics with friction coefficient γ = 1. Finally, a 100 ps molecular dynamics was completed at 310 K (Langevin dynamics), constant volume, and constant pressure with a hybrid Monte-Carlo barostat [81]. In (2), the membrane surface tension was set to 0 on the xy plane. Lastly, a mini (100 ps) molecular dynamics simulation of the previous conditions was completed, without any constraint on the system.

All equilibration steps and molecular dynamics simulation were carried out with an integration step of 2 fs. Non-bonded interactions were calculated with the particle mesh Ewald summation (PME), with a cut-off of 10 Å, and bonds involving hydrogen atoms were constrained with SHAKE algorithm [82]. The initial velocities were reassigned, according to the Maxwell-Boltzmann distribution, and the same parameters (simulation conditions) as the mini dynamics were applied. Coordinates were recorded every 1 ps. The system was simulated with AMBER18 (http://ambermd.org/AmberMD.php; accessed on 17 June 2021) using the PMED Cuda module, running of the supercomputer JEAN ZAY at IDRIS (http://www.idris.fr/jean-zay/, accessed on 17 June 2021). For system (1), a unique short trajectory of 1 ns and, for system (2), three extended trajectories of 2 µ were performed.

### 4.3. Data Analysis

All standard analyses were performed using the CPPTRAJ 4.25.6 program [83] of AmberTools20. Analysis of MD conformations (every 10 ps) was realized after least-square fitting on residues of the TK domain (W582-S688, L769-S931) or on residue-truncated trajectories of each fragment to remove rigid-body motions.

(1) The RMSD and RMSF values were calculated for the Cα-atoms, using the initial model (at t = 0 ns) as a reference. The RMSD, RMSF, and cross-correlations were calculated for the Cα-atoms on the initial conformation (t = 0 μs) as a reference.
(2) Secondary structural propensities for all residues were calculated using the define secondary structure of proteins (DSSP) method [34]. The secondary structure types were assigned for residues, based on backbone -NH and -CO atom positions. Secondary structures were assigned every 10 ps for the individual and concatenated trajectories, respectively.
(3) Clustering analysis was performed on the productive simulation time of each MD trajectory, using an ensemble-based approach [41]. The algorithm extracts representative MD conformations from a trajectory by clustering the recorded snapshots, according to their Cα-atom RMSDs. The procedure for each trajectory can be described as follows: (i) a reference structure is randomly chosen in the MD conformational ensemble, and all conformations within an arbitrary cut-off r are removed from the ensemble; this step is repeated until no conformation remains in the ensemble, providing a set of reference structures at a distance of at least r; (ii) the MD conformations are grouped into n reference clusters, based on their RMSDs from each reference structure. The cut-off was varied from 3 to 5 Å. The analysis was performed every 100 ps.
(4) The H-bonds between donor (D) and acceptor (A) atoms N, O, and S were monitored, according to the following geometrical parameters: d(D—A) ≤ 3.6 Å, $\hat{\mathrm{DHA}}$ ≥ 120°. Hydrophobic contacts were considered for all hydrophobic residues, with side chains within 4 Å of each other.
(5) The principal components analysis (PCA) modes were calculated for the backbone atoms (N, H, Cα, C, and O) after least-square fitting on the average conformation calculated on the concatenated data. The eigenvectors were visualized with NMWiz module for VMD [84].
(6) The normal modes and cross-correlation matrices of the average conformation of each replicate were calculated with the R library bio3D [75], at 310 K, with all available force fields.
(7) Curvature angles of selected secondary structures (helices or β-strand), relative to others or to their initial position (t = 0 μs), were calculated with Equation (1):
  (1) $$\Theta =\arccos\frac{v_{1}\cdot v_{2}}{\sqrt{∥v_{1}∥}\times \sqrt{∥v_{2}∥}}\;$$
  where Θ is the angle in radian and $v_{1}$ and $v_{2}$ the secondary structures representative vectors coordinates. The vectors where delimited in the N- to C-terminal directions on the Cα atoms of the most structurally stable residues, according the DSSP: P524−C537 (TMD), T594−A597 (β_1_ in P-loop), L637−G648 (αC helix), A701−N705 (αH1 in KID), S771−L783 (αE in C-lobe), and V824−K826 (β_9_ in A-loop). The kink angle of the TMD helix (P524−C537) was calculated according to [37], after least-square fitting of the TMD Cα on their initial conformation. Using the previous formula, the kink angle corresponds to the angle between the vector from the mid-point of hinge (V530) in the N-terminal direction and vector from V530 in the C-terminal direction.
(8) The radius of gyration (Rg) was calculated from the atomic coordinates using Equation (2) from [85]:
  (2) $$R_{g}=\sqrt{\frac{\sum_{i=1}^{N}m_{i}r_{i}^{2}}{\sum_{i=1}^{N}m_{i}}}$$
  where $m_{i}$ is the mass of the atom i and $r_{i}$ is the distance of atom i from the protein centre of mass.
(9) The relative Gibbs free energy of the canonical ensemble was computed as a function of two reaction coordinates with Equation (3) [86]:
  (3) $$\Delta G\;=-k_{B}T\;\ln\;\frac{P\left(R_{1},{\;R}_{2}\right)}{P_{\max}\left(R_{1},{\;R}_{2}\right)}$$
  where $k_{B}$ represents the Boltzmann constant, T is the temperature. $P\left(R_{1},R_{2}\right)$ denotes the probability density of states along the two reaction coordinates, calculated using their joint probability, and $P_{\max}\left(R_{1},R_{2}\right)$ denotes the maximum probability. The population of each well was roughly estimated using a square defined with $R_{1}$ and $R_{2}$ value intervals and containing red to orange ΔG colors.

### 4.4. Visualisation and Figure Preparation

Visual inspection of the conformations and figure preparation were performed with PyMOL (https://pymol.org/2/, accessed on 14 September 2020). The VMD 1.9.3 program [87] was used to prepare the protein MD animations. To visualise the motions along the principal components, the Normal Mode Wizard (NMWiz) plugin [84], which is distributed with the VMD program, was used. The three-dimensional representations of the free energy surface were plotted using Matlab (US, © 1994-2021 The MathWorks, Inc., Natick, MA, USA).

## Data Availability

The numerical model simulations, upon which this study is based, are too large to archive or transfer. Instead, we provide all the information needed to replicate the simulations. The model coordinates are available from L. Tchertanov at ENS Paris-Saclay.

## Supplementary Materials

The following are available online at https://www.mdpi.com/article/10.3390/ijms23031589/s1. References [42,88,89] are cited in the Supplementary Materials.
