# Supplementary material for: The Inherent Coupling of Intrinsically Disordered Regions in the Multidomain Receptor Tyrosine Kinase KIT
Source: Int J Mol Sci. 2022 Jan 29;23(3):1589. doi: 10.3390/ijms23031589 (PMC8835827; doi:10.3390/ijms23031589)
Supplement: Supplementary file 1 [file ijms-23-01589-s001.zip › ijms-1566029-supplementary.pdf]

Supplementary Material

The Inherent Coupling of Intrinsically Disordered Regions in the Multidomain Receptor Tyrosine Kinase KIT

Julie Ledoux, Alain Trouvé and Luba Tchertanov *

Centre Borelli, ENS Paris-Saclay, CNRS, Université Paris-Saclay, 4 Avenue des Sciences, F-91190 Gif-sur-Yvette, France

* Correspondence: luba.tchertanov@ens-paris-saclay.fr

Figures

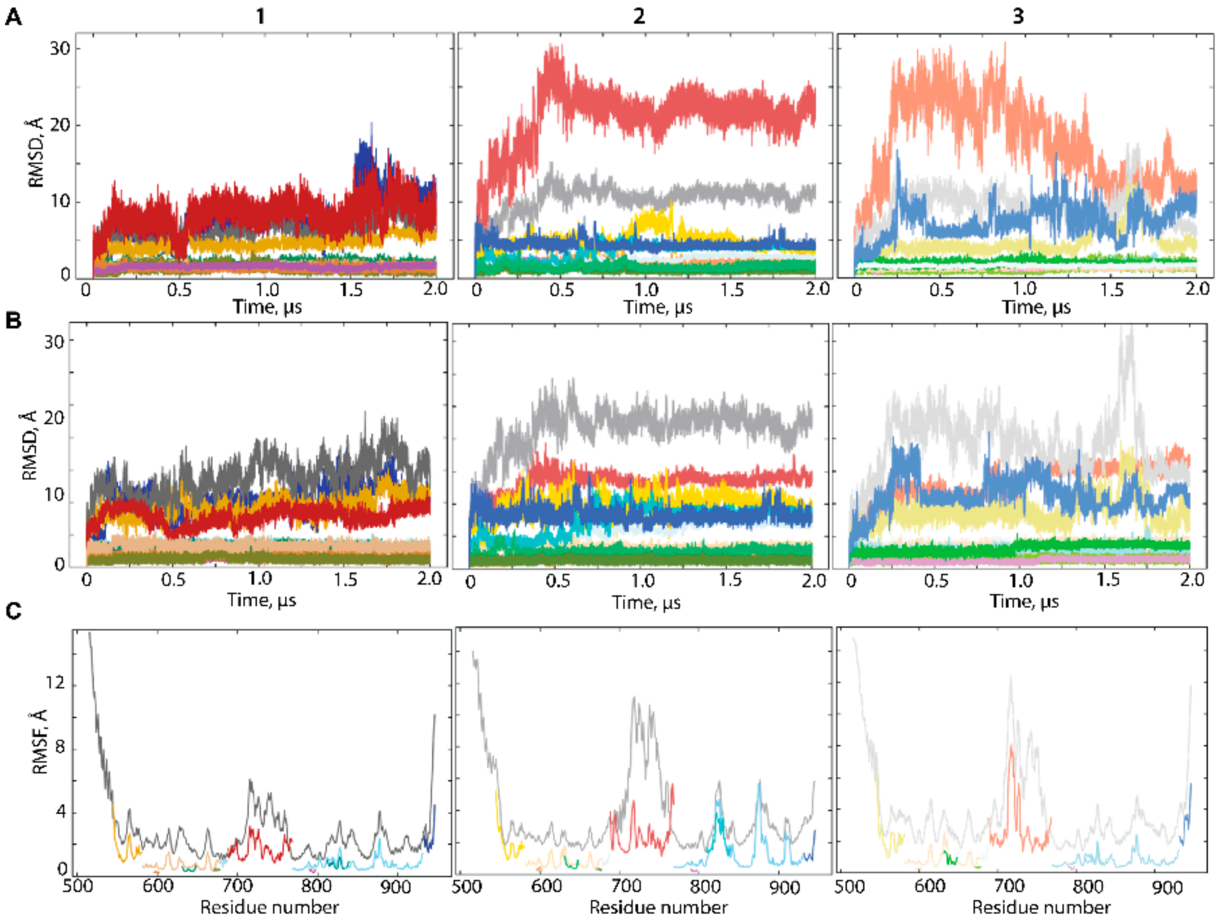

Figure S1. Molecular dynamics simulations of 3D model of the full-length cytoplasmic domain of RTK KIT in inactive state. (**A**,**B**) RMSDs from the initial coordinates (t=0 μs) computed for the Cα-atoms of the overall structure and individually for the Cα-atoms of KIT domain/regions after least-square fitting of the MD conformations of the TK domain on the initial conformations (**A**), and on the initial conformations of the respective domain (**B**). (**C**) RMSFs computed on the Cα atoms for MD conformations after the least-square fitting on the initial conformation of KIT or the respective domain. (**A**–**C**) KIT is in grey, N-lobe in beige, C-lobe in blue, JMR in yellow, P-loop in orange, αC-helix in green, hinge in olive, KID in red, C-loop in rose, A-loop in teal, C-tail in dark blue for the 1-3 trajectories (replicas) of MD simulations.

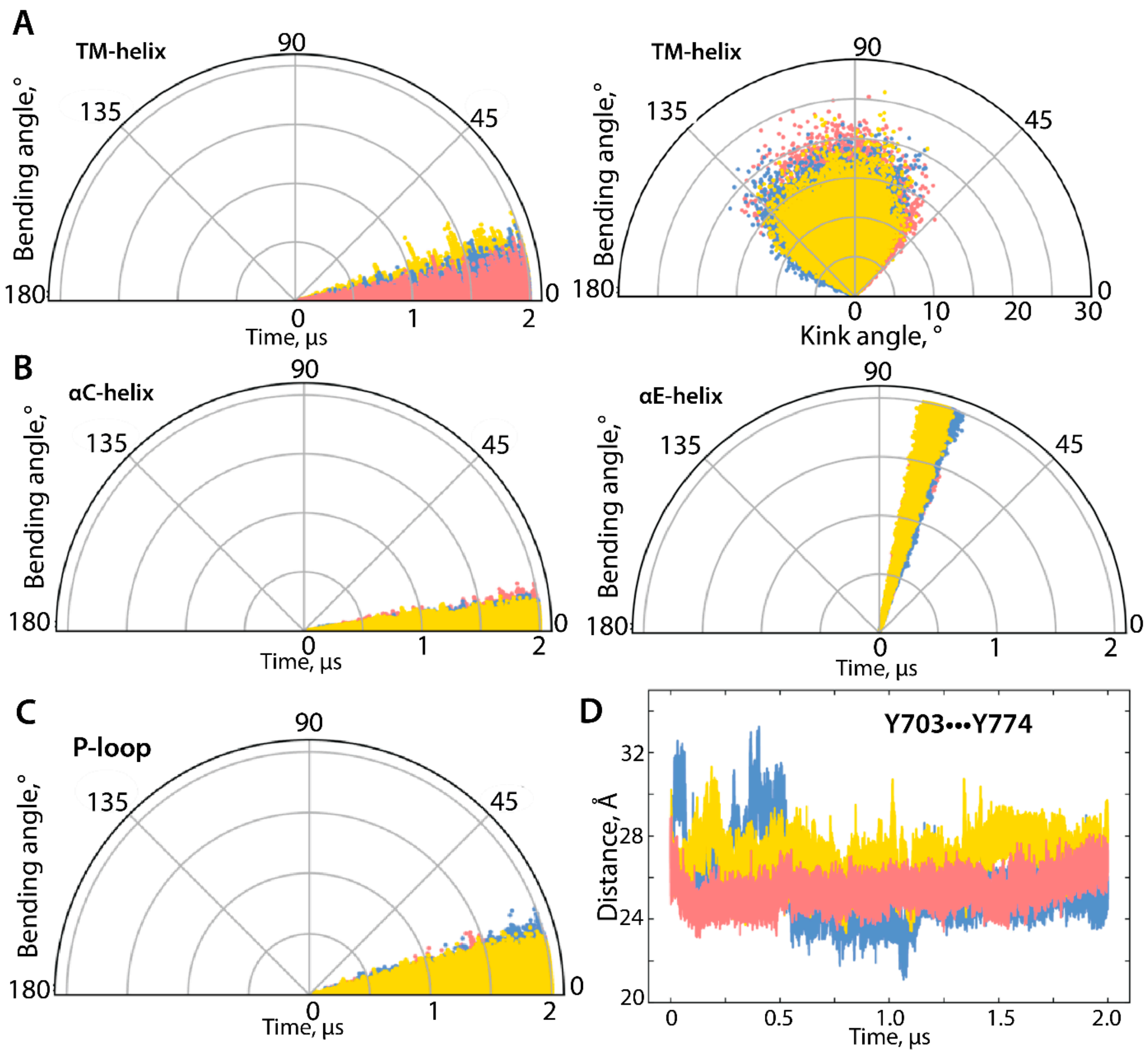

Figure S2. Geometry of the KIT fragments. (**A**) The angular variations of the TM helix, the bending angle (left) and distribution of the bending and kink angles (right). (**B**) Bending angle of the αC-helix (in respect to its staring conformation) and αE-helix (in respect to the membrane surface). (**C**) Bending angle of the P-loop. (**D**) Distance between the Cα-atoms of Y703 (αH1-helix of KID) and Y774 (αE-helix of the C-lobe). Calculations are performed after least-square fitting of the data on the TK domain. Conformations from different trajectories are distinguished by colour: red (1), blue (2) and yellow (3).

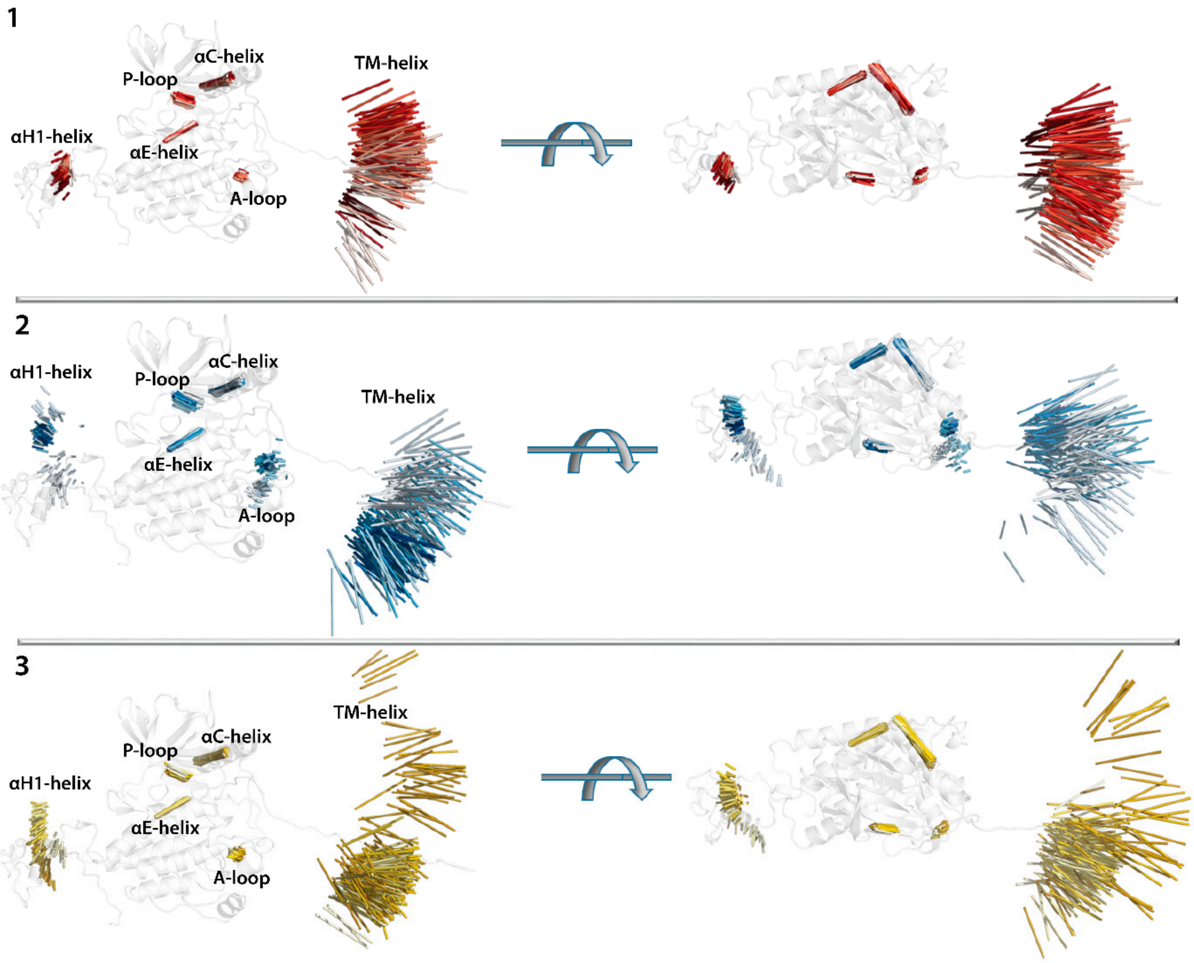

Figure S3. Conformational features of KIT. Positions of hints – TM-helix, αC-helix, αE-helix, αH1-helix, P-loop and β-hinge of A-loop – are superimposed on a mean conformation of KIT calculated each 10ps on the concatenated trajectories. Protein is shown as cartoon, each hint is presented by an axis of helix or by a vector colinear to a strand. Two orthogonal projections are shown. Calculations were performed on cMD conformations taken each 10 ns from the individual trajectories distinguished by colour – red (1), blue (2) and yellow (3). The colour gradient shows the evolution of a trajectory, from light (t = 0) to dark (t = 2 μs).

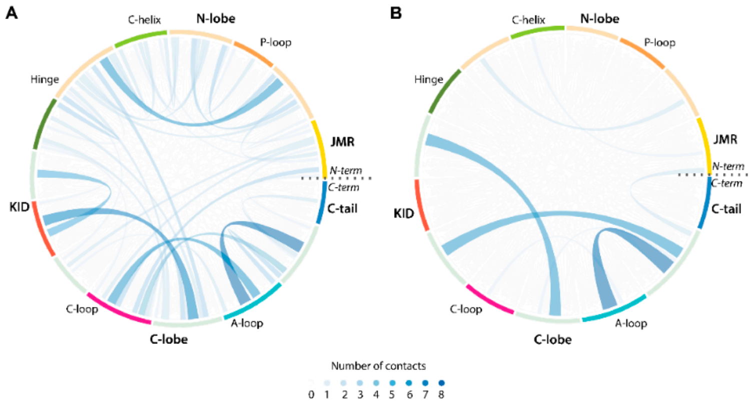

Figure S4. The non-covalent contacts stabilizing the crystallographic structure (PDB ID: 1T45) of RTK KIT in the inactive state**.** The string diagram compiles the H-bonds (**A**) and hydrophobic interactions (**B**) which are shown as curves coloured according to the occurrences of contacts, from 0 (white) to 8 (blue). The KIT domains and the functionnally related fragments are distinguished by colours and labelled in bold and regular font respectively. Contacts involved in the formation of regular structures (H-bonds forming helix or sheet) and intra-domain framework except the functionally related regions were excluded from consideration.

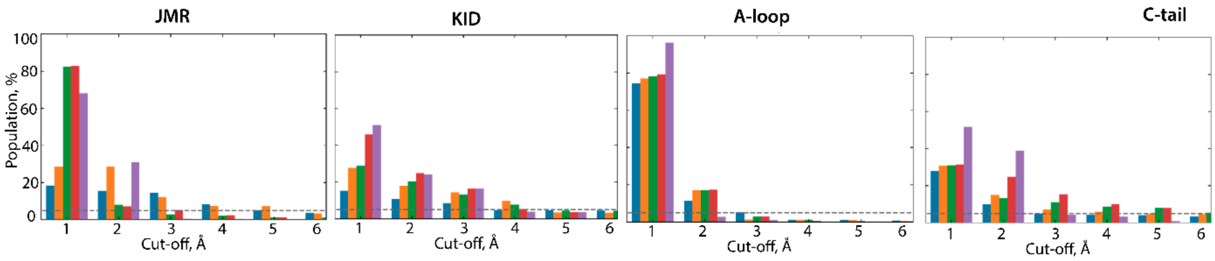

Figure S5. Ensemble-based clustering of cMD conformations of the highly varied KIT regions – JMR, KID, A-loop and C-tail. Percentage of the clusters population obtained for the concatenated trajectory. Clustering was performed each 100 ps of each trajectory using cut-off values varying from 2.0 to 4.0 Å, with a step of 0.5 Å. The lines in blue, orange, green, red, and violet correspond to cut-off of 2.0, 2.5, 3.0, 3.5 and 4.0 Å respectively. Cluster number above the grey dashed line represent cumulatively 95% of the population.

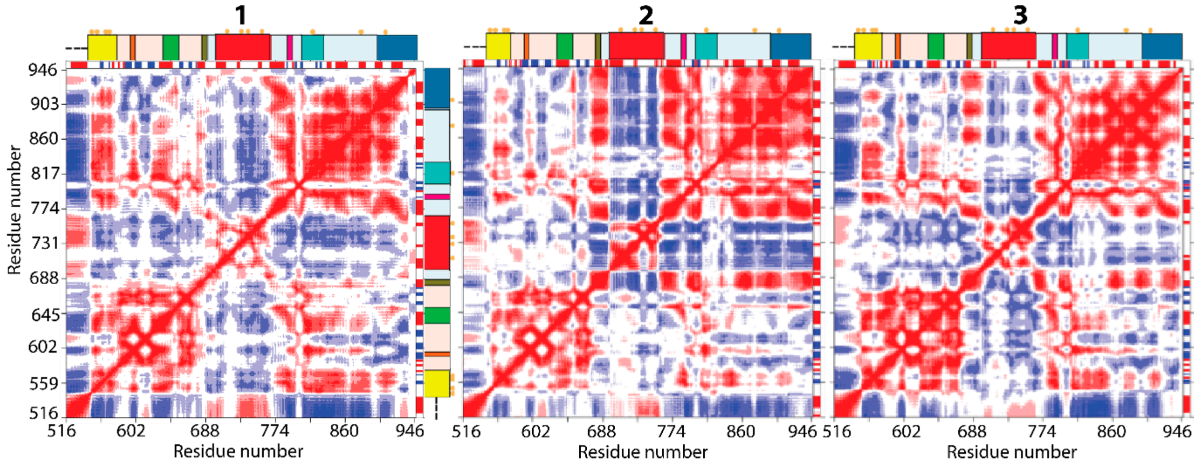

Figure S6. Intrinsic motion in KIT and its interdependence. Inter-residue cross-correlation map computed for the Cα-atom pairs of KIT computed for each cMD trajectory after least-square fitting on the initial conformation. Correlated (positive) and anti-correlated (negative) motions between Cα-atom pairs are shown as a red-blue gradient.

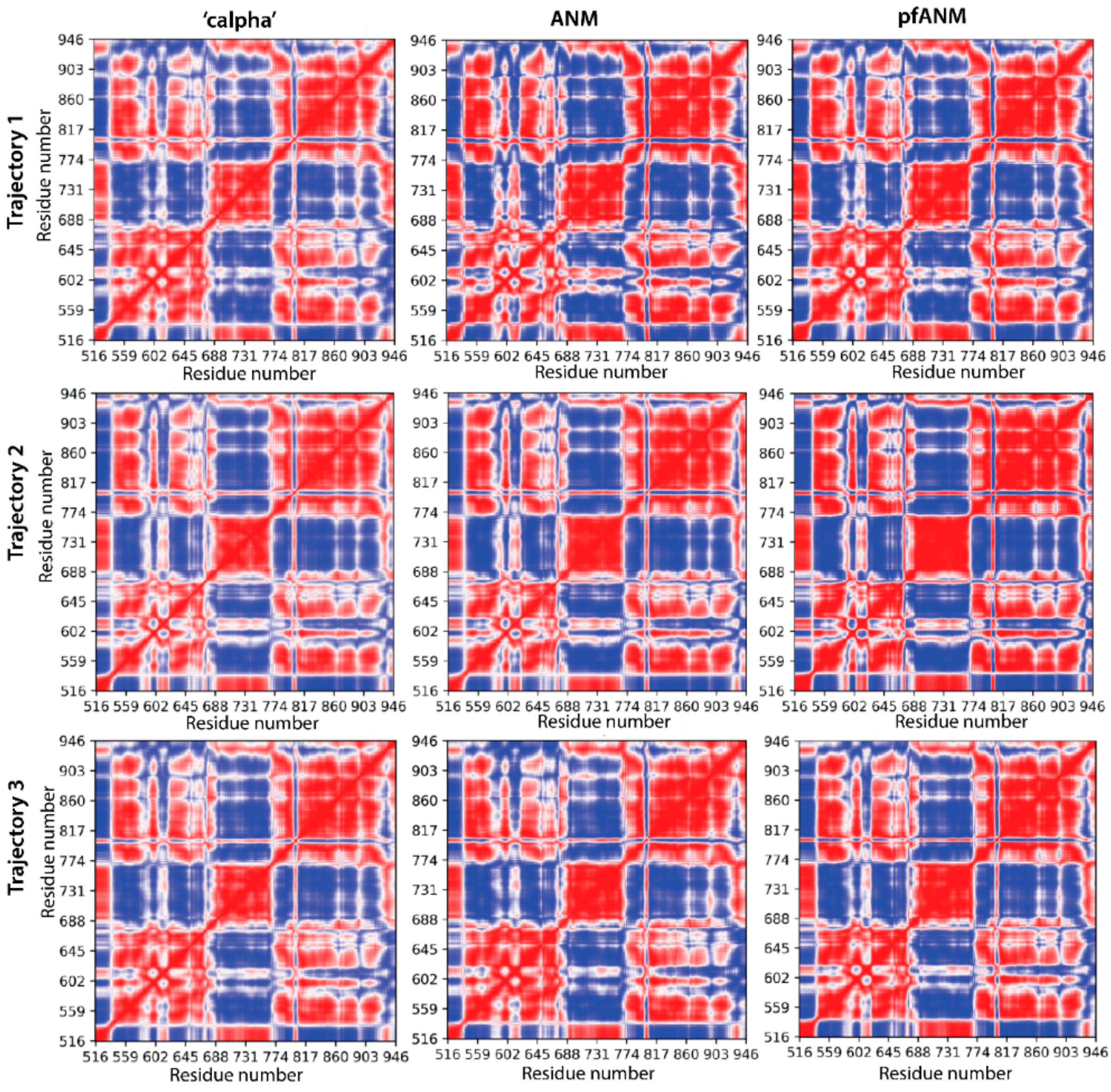

Figure S7. Intrinsic motion in KIT and its interdependence. Dynamical inter-residue cross-correlation maps resulting from NMA of the mean conformation of KIT in each cMD trajectory (1-3), calculated by using three different force fields – the 'capha', originally developed by [1], the Anisotropic Normal Model (ANM) [2] and Elastic Network model (pfANM) [3]. Correlated (positive) and anti-correlated (negative) motions between Cα-atom pairs are shown as a red-blue gradient.

References

1. Hinsen, K. Analysis of domain motions by approximate normal mode calculations. *Proteins Struct. Funct. Bioinform.* **1998**, *33*, 417–429. https://doi.org/10.1002/(SICI)1097-0134(19981115)33:3
2. Eyal, E.; Yang, L.-W.; Bahar, I. Anisotropic network model: systematic evaluation and a new web interface. *Bioinformatics* **2006**, *22*, 2619–2627, doi:10.1093/bioinformatics/btl448.
3. Yang, L.; Song, G.; Jernigan, R.L. Protein elastic network models and the ranges of cooperativity. *Proc. Natl. Acad. Sci. USA* **2009**, *106*, 12347–12352, doi:10.1073/pnas.0902159106.
